# Bioactive indanes: Development and validation of an LC-MS/MS bioanalytical method for the determination of PH46A, a new potential anti-inflammatory agent, in dog and rat plasma and its application to a pharmacokinetic study in dog

**DOI:** 10.1016/j.jpba.2019.113011

**Published:** 2020-02-05

**Authors:** Gaia A. Scalabrino, Tao Zhang, Neil Frankish, Helen Sheridan

**Affiliations:** a Trino Therapeutics Ltd, The Tower, Trinity Technology and Enterprise Campus, Dublin 2, Ireland; b School of Food Science and Environmental Health, City Campus, Technological University Dublin, Dublin 1, Ireland; c Drug Discovery Group, School of Pharmacy and Pharmaceutical Sciences & Trinity Biomedical Sciences Institute, Trinity College, Dublin 2, Ireland

**Keywords:** LC–MS/MS, PH46A, Rat plasma, Dog plasma, Validation

## Abstract

A new chemical entity, which is a chiral indane dimer, PH46A, has been developed by our research group. As a clinical candidate. PH46A has recently completed Phase I clinical studies in man. Previously, during its pre-clinical development, in *in vivo* pre-clinical studies PH46A showed potent anti-inflammatory properties, which can be targeted at a range of diseases, including inflammatory bowel disease (IBD). To support the pre-clinical development of this drug candidate, we developed a LC—MS/MS method for determining PH46 (the acid form of PH46A salt) in both dog and rat plasma using Compound 1 as internal standard (IS). Those species were selected for safety pharmacology and toxicology, as well as pharmacokinetics studies.

The method was validated over the range 10−10000 ng/mL for both matrices and the linearity, accuracy, precision and specificity over this range were demonstrated to be acceptable. No significant matrix effects or carryover were observed for both PH46 and IS and recovery was consistent. PH46 was found to be stable in both dog and rat plasma under the test conditions, such as at room temperature for >24 h, through 3 freeze/thaw cycles, and at -20 °C for >1 month. PH46 and IS in dog and rat plasma extracts were also found to be stable in the autosampler against fresh standard extracts on re-injection after 143.5 h and 243.5 h, respectively at 4 °C. 10- and 100-fold dilutions with control matrix were found not to affect the performance of the assay. This method was successfully applied to a pharmacokinetic study in the dog. With the exception of one dog, 003 M, oral administration of PH46A in gelatine capsules was well tolerated at a dose level of 100 mg/kg. The highest C_max_ was observed in animal 003 M. The rapid absorption and high plasma concentration observed for animal 003 M compared to the data for animals 001 M and 002 M may account for the sickness observed in this animal; however, the reasons for this have not been investigated.

## Highlights

• Selective and sensitive determination by LC—MS/MS of PH46A, a new class of antiinflammation agent, in preclinical animal plasma.
• Full development and validation of the LC—MS/MS method in two preclinical species.
• A pharmacokinetic study of PH46A in dog using the method developed.

## Graphical abstract

**Figure:**
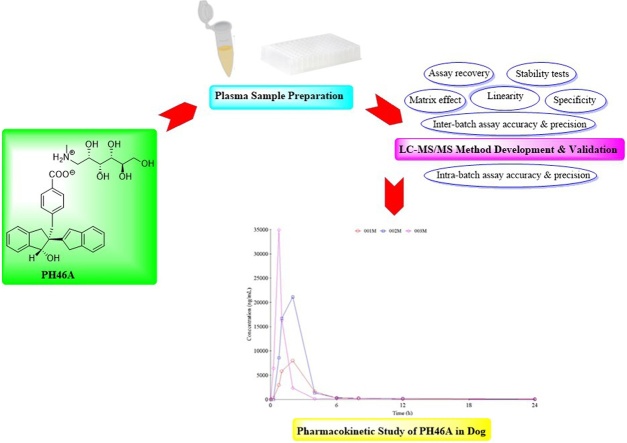

## 1 Introduction

Our research group have developed a series of indane dimers and the biological activities of these molecules have been investigated [[1], [2], [3], [4], [5], [6]]. During the course of our work, a novel indane scaffold was discovered which demonstrated potential treatment of inflammatory conditions, in particular inflammatory bowel disease (IBD) [7]. A lead, first-in-class molecule, PH46A, 6-(methylamino)hexane-1,2,3,4,5-pentanol-4-(((1*S*,2*S*)-1-hydroxy-2,3-dihydro-1H,1′H-[2,2-biinden]-2-yl) methyl) benzoate [8,9] has been shown to have a biological effect in two different well-established preclinical models of murine colitis: the acute dextran sodium sulphate model and the chronic and spontaneous Interleukin-10 (IL-10^−/−^) knock-out mouse model [8]. This is indicative that PH46A has a therapeutic effect which is independent of model specific aetiology. Based on the effects in the animal models, PH46A is believed to be a potential new treatment for patients with IBD and it has recently completed a Phase I clinical trial study in healthy volunteers [10].

**Fig. 1:**
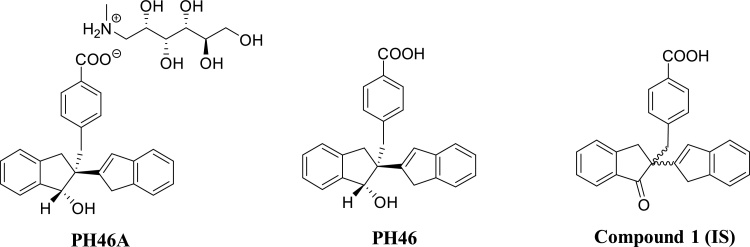
Chemical structures of PH46A, PH46 and Compound 1 (IS).

The *in vitro* comparative metabolism study of PH46A that we recently published showed that PH46A metabolism in rat is most similar to human, and all putative human metabolites are present in rat, dog, mouse and cynomolgus monkey [11]. Rat and dog were therefore selected as appropriate and useful species for use in subsequent mandatory and pivotal GLP safety pharmacology and toxicology studies in order to guide the clinical development of PH46A. Further, at this time, studies in laboratory animals provide the best available basis for extrapolation to humans and are required to support regulatory submissions. Acceptable models which do not use live animals currently do not exist. The rat was selected as the test model, since it is accepted by regulatory authorities as a rodent animal model for toxicity studies [12], high quality animals are readily available, and a large amount of background pathology data is available in this species. The dog was also selected as the test model to meet regulatory requirements for testing in a non-rodent species [12] and because of the availability of background data and the proven suitability in toxicology studies. As part of the preclinical investigations, the characterisation of the pharmacokinetics (PK) of PH46A in the rat and the dog were studied. To this end, this manuscript describes the development and validation of sensitive and specific LC–MS analytical methods for determining PH46 (the free acid form of PH46A salt) (Fig. 1) in dog and rat plasma according to the Food and Drug Administration (FDA) [13] and the European Medicines Agency (EMA) [14] guidelines. The method was subsequently applied to a PK study in male Beagle dogs to quantitate levels of PH46 and evaluate its oral bioavailability following oral administration of PH46A in capsule.

## 2 Materials and methods

### 2.1 Chemical and reagents

PH46A [purity 97.1 %; correction factor (purity and salt content): 0.6429] and Compound 1 as Internal Standard (IS, Fig. 1) (purity 99.8 %) were obtained from Trino Therapeutics Ltd (Ireland). Control rat (Sprague Dawley) and dog (Beagle) plasma and blood were obtained from Charles River Laboratories (UK) using lithium heparin as anticoagulant and stored at -20 °C when not in use (all control plasma was mixed and centrifuged prior to use). HPLC grade solvents and additives, including methanol (MeOH), acetonitrile (ACN), water, ammonia solution, acetic acid (AA), acetone, dimethylsulphoxide (DMSO), chloroform and formic acid (FA), were purchased commercially between VWR (Ireland) and Sigma-Aldrich (Ireland) and were used without further purification. Size 12 gelatine capsules (Torpac ‘Loc Ring Capsules’, USP grade) were used to administer the dose for the pharmacokinetic study.

### 2.2 Animals and husbandry

All animal research work reported in this article have been carried out in accordance with Directive 2010/63/EU and its Irish transposition S.I No 543 of 2012. Ethical approval was obtained through Trinity College Dublin which complies with the Council for International Organizations of Medical Sciences’ (CIOMS), International Guiding Principles for Biomedical Research Involving Animals, and all laws, regulations and policies governing the care and use of laboratory animals in the jurisdiction in which the research is being conducted. Three male Beagle dogs (on-study numbers: 001 M, 002 M and 003 M, respectively) body weight 9.9–11.2 kg, were supplied by Charles River Laboratories (UK). During pre-trial and on-study periods, the animals were gang housed in cages appropriate to the species. Prior to inclusion on this study, animals were subject to a veterinary examination and the results found to be satisfactory. All animals were weighed prior to each dose administration and body weights recorded. Animals were fasted overnight prior to dosing and offered standard laboratory diet (PM1 Lab Diet 5007, Purina) for 2 h post-dose. Mains quality tap water was available *ad libitum* throughout the study.

### 2.3 Instrumentation and operation conditions

AB Sciex API 3000 LC/MS/MS System consisted of HPLC pump (Series 200, Perkin Elmer), autosampler (HTS Pal, CTC Analytics), column oven (Series 200, Perkin Elmer) and six-port switching valve (Valco). Operations and data analysis were controlled by data handling system (Analyst Version 1.4.2, AB Sciex) and Laboratory Information Management System (Watson 7.0, Thermo Fisher Scientific). Halo C18 column (75 × 2.1 mm, 2.7 μm, HiChrom) and PreFrit Filter guard column (0.5 μm, Anachem) was employed at 60 °C with autosampler temperature at 4 °C. Mobile phase A: MeOH:AA (100:0.2, v/v) and mobile phase B: water:AA (100:0.2, v/v). The gradient was 40 % B (0 min), 20 % B (2.0 min), 20 % B (4.0 min), 0 % B (4.5 min), 40 % B (4.7 min), 0 % B (4.9 min) and 40 % B (8.0 min). Flow rate was 0.3 mL/min with injection volume of 10 μL and run time of 8 min. The solvent mixtures for needle wash are MeOH/water/acetone/FA (40/40/20/1, v/v/v/v) and MeOH/water/ammonia (50/50/1, v/v/v).

Mass spectrometric detection was performed using TurboIonSpray negative ionization mode. Standard API3000 nitrogen was used for nebulizing and drying. Ion spray temperature was 550 °C and ion spray voltage was −4500 V. The probe positions were 5 mm (X) and 5 mm (Y). PH46 ion monitored was 381.10 → 135.10 (± 0.5) dwell 150 msec and IS ion monitored was 379.10 → 134.14 (± 0.5) dwell 150 msec.

### 2.4 Preparation of control matrix

The haemolysed plasma was prepared by overtaxing and freezing an aliquot of the blood at −20 °C for a short period of time. This was then thawed, centrifuged (3500 rpm, 4 °C & 10 min) and the resultant plasma was transferred to a clean tube for use. All control plasma was stored at −20 °C when not in use and was mixed and centrifuged prior to use.

### 2.5 Preparation of calibration standards and quality control samples

The primary stock solutions of PH46 and IS were prepared at 1000 μg/mL in ACN/DMSO (50:50, v/v). The calibration standards (CSs) were prepared by serially diluting the primary PH46 stock solution in control matrix to give concentrations at 10, 20, 65, 250, 750, 2500, 9000 and 10000 ng/mL plasma. For each batch of samples, aliquots (50 μL) of CSs were extracted in duplicate to give a range of concentrations of PH46 within the linear range of the assay and a fixed concentration of IS (300 ng/mL) prepared from IS stock using MeOH/chloroform (50:50, v/v). Quality control (QC) samples were prepared in control matrix from the primary PH46 stock solution in similar manner as CSs, to give QCs at 10 [Lower Limit of Quantification (LLOQ)], 25 (low), 300 (medium) and 8000 (high) ng/mL plasma concentrations and aliquots (50 μL) of QCs were extracted for analysis. All the solutions were stored at −20 °C and brought to room temperature before being analysed. A correction for batch specific purity was applied to all weighing, as well as a correction for salt content (382.5/577.7; free acid/salt).

### 2.6 Sample extraction

Control plasma sample (Lithium Heparin anticoagulant) was removed from the freezer and allowed to thaw at room temperature. Control matrix was vortex mixed and centrifuged prior to use. 50 μL was added to each appropriate well of a clean round bottomed 96 well plate for double blank (DB) (control matrix only without PH46/IS) and single blank (SB)control matrix with IS only) samples. CSs, QCs and test samples were removed from the freezer and allowed to thaw at room temperature and vortex mixed prior to aliquoting. 50 μL was added to each appropriate well of a clean round bottomed well plate. 50 μL of IS [0.3 μg/mL in MeOH/chloroform (50:50, v/v)] was added to all samples except for DBs, which received 50 μL of MeOH/chloroform (50:50, v/v). A further 500 μL of MeOH/chloroform mixture was added to all samples and the plate was sealed and vortex mixed thoroughly prior to centrifugation (3500 rpm, 5 min, 4 °C). The supernatant from all samples was transferred into clean matrix tubes, dried under nitrogen at 60 °C and then reconstituted in 200 μL of MeOH/water/AA (50:50:0.2, v/v/v). The extracts were vortex-mixed and centrifuged for 5 min at 3500 rpm at 4 °C.

### 2.7 Validation procedures

The analytical method was developed and validated for use on the Tomtec robotic pipetter using 96 well plates and matrix tubes in a 96 well format. Since the test sample PH46A was weighed and a correction factor was use for the salt content, all peak area measurement and determined concentrations reported were for PH46 acid. Each batch of samples consisted of matrix DB, matrix SB, CSs, QC and test sample extracts. One set of CSs was injected at the start of the run and one set at the end of the run. CSs were injected in concentration order in each section of the run. QC samples were prepared in control matrix at low, medium and high levels (n≥2 at each level). Where n = 2, one low & one medium level QC samples were injected at the start of the run after one set of CSs, and one low & one high level QC samples were injected at the end of the run before the other set of CSs. The remaining medium & high QCs were injected midway through the run. Where n>2, the additional QCs were appropriately distributed throughout the run. Solvent or extracted matrix DBs were injected after high concentration extracts prior to injecting a potentially low concentration extract in order to avoid the risk of assay carryover. The target acceptance criteria for QCs was that the determined concentrations of at least 66 % of QCs in each batch had to be within 100 ± 15 % of the nominal concentrations, including at least one QC sample at each concentration had to meet this criterion.

#### 2.7.1 Stability of PH46 and IS in stored stock solutions

The effect of PH46 storing stock solutions in ACN/DMSO (50/50, v/v) at 4 °C was investigated by comparison of peak area ratios of freshly weighed stock solutions with stock solutions which had been prepared 35 days previously. Both stock solutions were prepared in the same way. The stock solutions were diluted to the one upper limit of quantification (ULOQ) level with an appropriate diluent. The effect of storing solutions of IS (working and stock solutions) in ACN/DMSO, (50/50, v/v) for the stocks and MeOH/chloroform (50/50, v/v) for the working solution at 4 °C was investigated by comparison of peak area ratios of freshly weighed and diluted stock and working solutions with solutions which had been prepared 35 (stocks) and 27 (working solutions) days previously. PH46 and IS were deemed to be stable if the mean detector responses for the stored solutions were within 100 ± 10 % of the mean detector responses of the freshly prepared solutions and the precision is ≤10 %. The effect of storing stock solutions of PH46 in ACN/DMSO (50/50, v/v) at ambient room temperature was also studied by comparison of peak area ratios of an aliquot of the stock solution stored at 4 °C with an aliquot of the same stock solution stored at ambient room temperature for 24 h. Both solutions were diluted to the ULOQ level with an appropriate diluent. PH46 was expected to be stable at room temperature if the mean detector responses for the solutions stored at room temperature were within 100 ± 10 % of the mean detector responses of the solutions stored at ca 4 °C and the precision is ≤10 %.

#### 2.7.2 Specificity and linearity

The assay specificity was determined by extraction and analysis of six different sources of the matrix (different batches of matrix were pooled from different animals). For each source, the samples were prepared, including one DB (control matrix only), one SB (control matrix + IS), one LLOQ (IS only) and ULOQ (without IS). If interfering peaks at the retention times (RTs) of PH46 or IS were noted, these were deemed to be insignificant if the response at RT of PH46 was ≤20 % of the response in LLOQ samples and if the response at the RT of IS was ≤5 % of the IS response. The 16-point calibration curves over 10−10000 ng/mL were constructed by plotting the peak area ratio of PH46:IS against the nominal matrix concentrations of PH46 to determine the optimum regression parameters. The matrix concentration of PH46 from each of CSs was calculated from the corresponding curve. A DB sample and a SB sample were also extracted and analysed, these were not included in the regression analysis. The acceptance criteria were that at least 75 % [a minimum of 6 including at least one LLOQ and one ULOQ sample] of the standards had to be back-calculated to be within 100 ± 15 % of the nominal concentrations (100 ± 20 % at LLOQ). Confirmation of linearity was performed at least on three separate occasions during the validation.

#### 2.7.3 Assay recovery and matrix effect

The matrix effects were determined by extracting replicate (n = 6) samples of control matrix, from six individual sources (one of these sources was haemolysed plasma which matched one of the non-haemolysed sources), and PH46 & IS solutions in MeOH/water/AA (50:50:0.2, v/v/v) were spiked after extraction. This resulted in replicate matrix effect samples (n = 3) at each of the matrix equivalent PH46 concentrations of 25 and 8000 ng/mL and IS concentration of 3000 ng/mL. Replicate (n = 3) non-extracted QC samples were prepared in the same ratio as the extract samples. The matrix factor (MF) for PH46 and IS was determined in each source of matrix by calculating the ratio of the mean response of replicate samples spiked into matrix to the mean response of the non-extracted samples. The IS-normalised MF was calculated by dividing PH46-MF by IS-MF from the same source. No matrix effect in the samples was expected if the precision of the IS-normalised MF calculated from the six sources was ≤15 %. The assay recovery was determined by preparing, extracting and analysing replicate (n = 3 at low, medium and high levels) matrix effect samples from a single source of matrix (spiked after extraction). Replicate (n = 3) extracted samples were prepared, extracted and analysed as detailed in Section 6.10 at the same low, medium and high concentrations as the matrix effect samples. The recoveries of PH46 and IS were defined as the mean responses in the extracted samples/the mean responses in the matrix effect samples.

#### 2.7.4 Intra- and inter-batch assay accuracy and precision

The assay intra- and inter-batch accuracy and precision were evaluated using replicate (n = 6) QC samples at 10 (LLOQ), 25 (low), 300 (medium) and 8000 (high) ng/mL. The intra- and inter accuracy expressed as the mean percentage determined concentration/nominal concentration, which was expected to be within 100 ± 15 % at each concentration level (100 ± 20 % at LLOQ level). Intra- and inter-precision were determined by the coefficient of variation of the mean determined concentration, which should be ≤15 % at each level (≤20 % at LLOQ level). The intra-batch assay accuracy and precision were determined on three occasions and a different batch of mobile phase was used on each occasion; three batches at each level were used for inter-batch assays.

#### 2.7.5 Assay carryover and matrix dilution

Carryover was assessed by using replicate QCs (n = 2) at LLOQ & ULOQ levels and additional replicate (n = 2) DBs. The sequence of analysis was: 1 x ULOQ, 2 x DBs, 1 x LLOQ, 1 x ULOQ, 2 x solvent samples (SSs) (MeOH/water/AA, 50:50:0.2, v/v/v) and 1 x LLOQ. No assay carryover for PH46 can be concluded if the PH46 responses in DB and SS were ≤20 % of the detector responses for PH46 in the following LLOQ samples; no IS carry-over if the IS responses in DB and SS were ≤5 % of the detector responses for IS in the preceding ULOQ samples. Replicate (n = 6) QC samples were made by diluting 10- and 100-fold of a bulk stock (80000 ng/mL in control matrix). Aliquots (50 μL) (n = 6 of each dilution factor) were then extracted (nominal concentrations of 8000 and 800 ng/mL). The determined concentrations were corrected for the dilution factors. Matrix dilution was considered to be acceptable if the assay accuracy was within 100 ± 15 % and the assay precision was ≤15 %.

#### 2.7.6 Stability experiments

Six aliquots (50 μL) of QCs at each low (25 ng/mL) and high (8000 ng/mL) concentration levels were extracted and analysed to confirm the suitability of the stability samples. Freeze/thaw stability of PH46 in matrix was performed using replicate QCs samples (n = 6) at each concentration level receiving three freeze/thaw cycles at −20 °C (initial freeze cycle at least 24 h, cycles 2 and 3 at least 12 h and thaw cycle for an hour at room temperature).

Ambient room temperature stability of PH46 in matrix was assessed on further QCs (n = 6) at each level after left at ambient temperature for 24 h before extraction with fresh CSs and QCs.

Short term matrix frozen storage stability was achieved by analysing replicate QCs (n = 6) at each concentration after one month’s storage at −20 °C.

Low and high QC samples extracted and previously injected for accuracy and precision batches (occasion two for dog plasma and occasion one for rat plasma) were re-injected for autosampler stability. The extracts had been stored at 4 °C for 143.5 h and 243.5 h for dog and rat batches respectively.

Reinjection stability was assessed by using the occasion 1 accuracy and precision batches, having stored the extracts at 4 °C for 131.5 h and 129.5 h for dog and rat batches respectively.

All stability samples were processed and quantified against freshly spiked CS and freshly spiked QC samples. The samples were considered to be stable if the mean determined concentration/nominal concentration was within 100 ± 15 % and the coefficient of variation was ≤15 % at each concentration level for each stability experiment.

### 2.8 Data processing and calculations

Data collection carried out during this study was performed using Analyst Version 1.4.2 data handling software. Determination of the calibration parameters (slope, intercept and coefficient of determination) and the calculation of mean, standard deviation and accuracy data were performed using Thermo Fisher Scientific Watson™ Version 7.0 software. The nominal matrix concentrations of CS and QC samples were entered to three significant figures. Determined concentration data are determined to three significant figures. For the CS and QC sample data, mean values were calculated from the unrounded determined concentration data and are presented rounded to three significant figures. Accuracy data were calculated from the rounded mean values and are presented to one decimal place. Precision data were calculated from the unrounded determined concentration data and peak area data and are presented rounded to one decimal place. Slopes and intercepts are presented to four significant figures; coefficients of determination are presented to four decimal places. Peak area ratios are presented to six decimal places.

### 2.9 Pharmacokinetic study in dog

The PH46A capsules were prepared the day before the dosing by weighing a suitable amount of PH46A into each capsule to achieve a target dose level of 100 mg/kg. The capsule fill weights were 1077.4 mg, 1119.6 mg and 990.4 mg for Capsule 1 (001 M), Capsule 2 (002 M) and Capsule 3 (003 M), respectively. Each capsule was administered by placing it in the back of the throat of each animal followed by swallowing and visual confirmation that the capsule was swallowed. Following dosing, blood samples (1 mL) were collected from the jugular vein into tubes containing lithium heparin as an anticoagulant at the following sampling times: pre-dose, 0.25, 0.75, 1, 2, 4, 6, 8, 12 and 24 h post-dose. Plasma was separated by centrifugation (3000 rpm, 10 min & 4 °C) prior to storage at −80 °C until analysis. Individual PH46 plasma concentrations generated were used to estimate pharmacokinetic parameters using WinNonLin v4.1 pharmacokinetic software.

## 3 Results and discussion

### 3.1 Stability of PH46 and IS in stored stock solutions

Both PH46 and IS stock solutions were found to be stable for at least 35 days at 4 °C, IS working solutions for at least 27 days at 4 °C for, and PH46 stock solutions for at least 24 h at room temperature.

### 3.2 Specificity and linearity

**Fig. 2:**
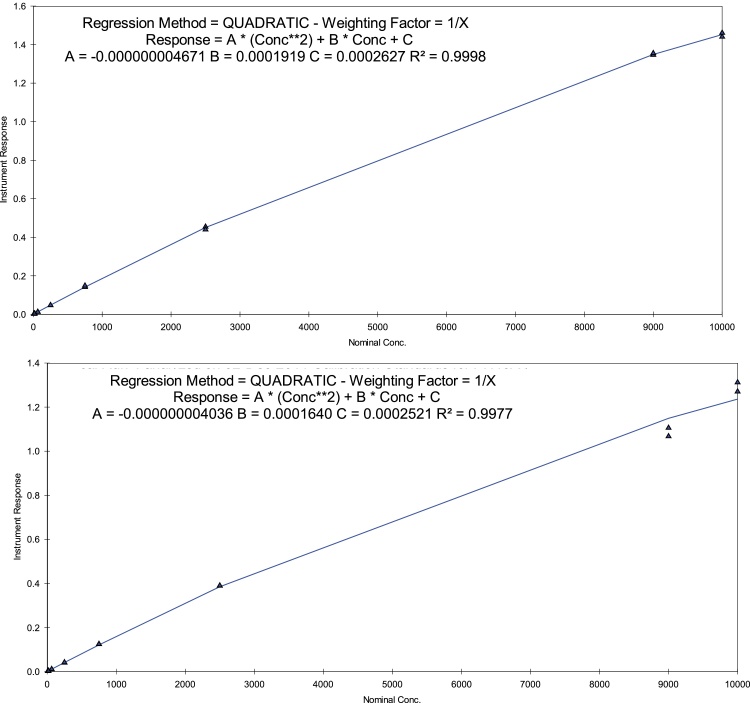
Calibration curves for the determination of PH46 in dog (upper) and rat (lower) plasma.

The calibration data from both dog plasma (8 batches) and rat plasma (7 batches) standards (over a range of 10−10000 ng/mL, duplicate run at each concertation) were analysed. In all cases at least 75 % of standards (and a minimum of 12 standards, including at least one replicate at the LLOQ and ULOQ levels) used to construct each calibration line met the acceptance criterion of the determined concentrations being within 100 ± 15 % of the nominal concentrations (100 ± 20 % of the nominal concentration at the LLOQ). Regression analysis of the peak area ratios of PH46:IS against the concentration demonstrated good linearity for dog and rat plasma over the range 10−10000 ng/m using a quadratic regression with a weighting factor of 1/x. For PH46 in dog plasma, the mean values of the regression equation y = Ax^2^+Bx + C were A: -0.000000007620, B: 0.0003271, C: 0.0002245 and R^2^ (coefficient of determination): 0.9986 for Occasion 1; A: -0.000000004671, B: 0.0001919, C: 0.0002627 and R^2^: 0.9998 for Occasion 2 and A: -0.000000004328, B: 0.0002098, C: 0.0005037 and R^2^: 0.9991 for Occasion 3, where y: the peak-area ratio of PH46 to IS; x: the plasma concertation of PH46. For PH46 in rat plasma, A: -0.000000004036, B: 0.0001640, C: 0.0002521 and R^2^: 0.9977 for Occasion 1; A: -0.000000005498, B: 0.0002251, C: 0.0007108 and R^2^: 0.9996 for Occasion 2; A: -0.000000004418, B: 0.0002091, C: 0.0003914 and R^2^: 0.9941 for Occasion 3. All determined concentrations for these standards met the acceptance criteria. The representative calibration curves from Occasion 2 are presented in Fig. 2.

**Fig. 3:**
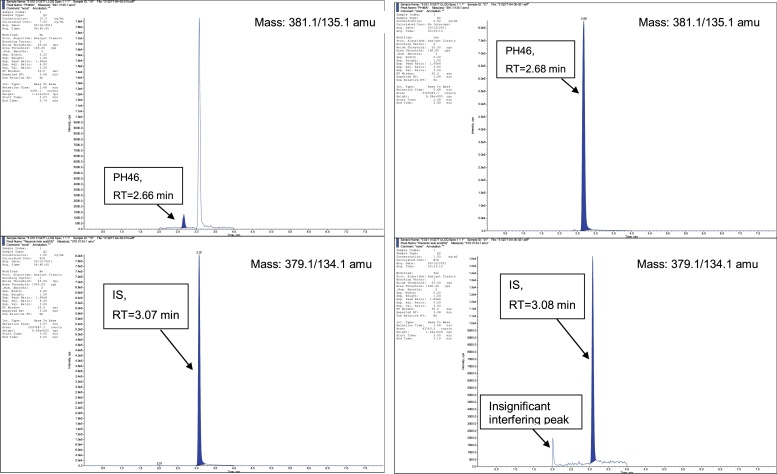
Chromatograms of a dog plasma extract (left) containing PH46 (LLOQ, 10 ng/mL) & IS (3000 ng/mL) and a dog plasma extract (right) containing PH46 (ULOQ, 10000 ng/mL) & no IS.

**Fig. 4:**
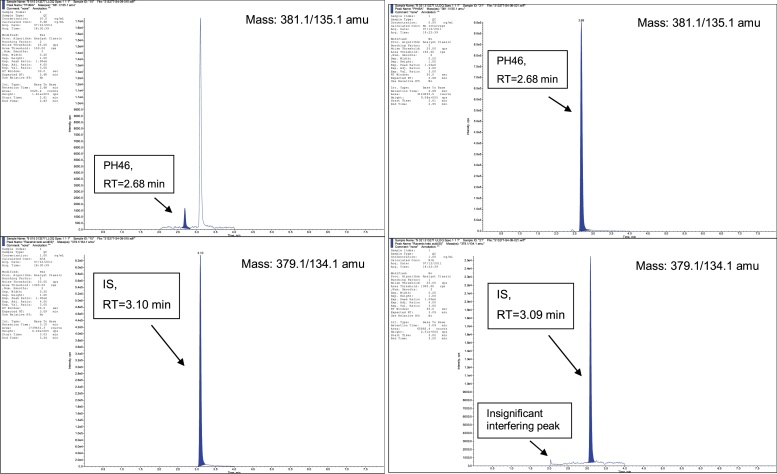
Chromatograms of a rat plasma extract (left) containing PH46 (LLOQ, 10 ng/mL) & IS (3000 ng/mL) and a rat plasma extract (right) containing PH46 (ULOQ, 10000 ng/mL) & no IS.

The QC sample data for the supporting QC samples are presented in Table 1, which met the acceptance criteria of the determined concentrations being within 100 ± 15 % of the nominal concentration. The assay specificity demonstrated there were no significant interfering substances at the RTs of PH46 and IS. A peak at RT of IS was observed in the PH46 chromatograms in samples containing IS due to in-source breakdown of IS. This did not interfere with the PH46 peak as both peaks were well resolved. A peak was also observed at RT of IS in samples containing only PH46, however, it was ≤5 % of the IS response and was therefore within acceptable limits. Representative chromatograms of dog plasma and rat plasma extracts are shown in Fig. 3, Fig. 4, respectively.

**Table 1:** Quality control data relating to PH46 in dog and rat plasma (results expressed as ng/mL).

	PH46 in dog plasma			PH46 in rat plasma		
	25.0*	300*	8000*	25.0*	300*	8000*
Batch 7	24.4	319	8120	–	–	–
	22.9	316	8730			
Batch 12	22.9	298	9130	–	–	–
	27.7	330	8550			
Batch 17	24.1	337	8100	–	–	–
	25.7	330	8610			
Batch 18	–	–	–	24.2	306	7930
				23.2	302	8480
Mean	24.6	322	8540	23.7	304	8210
Precision (%)	7.5	4.3	4.6	n/a	n/a	n/a
Accuracy (%)	98.5	107.2	106.8	94.8	101.3	102.6

* Nominal concentration of PH46. Precision: coefficient of variation of mean. Accuracy: mean determined concentration/nominal concentration. n/a: not applicable.

### 3.3 Assay recovery and matrix effects

For PH46 and IS in dog and rat plasma, the recoveries were consistent (Table 2). For PH46 and IS in dog plasma, at PH46 of 25 ng/mL the IS-normalised MFs from all six sources were 1.094, 1.131, 1.149, 1.111, 1.198, 1.071 and the corresponding precision was 4.0 %. At 8000 ng/mL level, the MFs were 1.248, 1.296, 1.312, 1.250, 1.382, 1.357 and the associate precision was 4.2 %. For PH46 and IS in rat plasma, the MFs at PH46 of 25 ng/mL were found to be 1.079, 1.028, 1.067, 1.054, 1.122, 1.029 and the precision was 3.3 %. At 8000 ng/mL level, the MFs were 1.383, 1.262, 1.273, 1.263, 1.358, 1.261 and the precision was 4.3 %. These results demonstrated that the matrix effects met the acceptance criteria and no significant matrix effect was present for PH46 and IS in both dog and rat plasma by the current method.

**Table 2:** Recoveries from dog and rat plasma samples of PH46 and IS (results expressed as peak areas).

	PH46 in dog		IS in dog		PH46 in rat		IS in rat	
	extracted samples	matrix extracts	extracted samples	matrix extracts	extracted samples	matrix extracts	extracted samples	matrix extracts
25.0 ng/mL*Mean Recovery (%)	n = 3 25490.6	n = 3 29225.5	n = 3	n = 3	n = 3 33549.4	n = 3 33775.9	n = 3	n = 3
	87.2		n/a		99.3		n/a	
300 ng/mL*Mean Recovery (%)	n = 3 309814.5	n = 3 361785.4	n = 3	n = 3	n = 3 373108.5	n = 3 422655.8	n = 3	n = 3
	85.6		n/a		88.3		n/a	
8000 ng/mL*Mean Recovery (%)	n = 3 6517584.8	n = 3 7452637.7	n = 3 4530866.7	n = 3 5099520.4	n = 3 7633624.1	n = 3 6912293.9	n = 3 6205135.6	n = 3 5505430.4
	87.5		88.8		110.4		112.7	

*Nominal concentration of PH46. Extracted samples = PH46 and IS extracted from plasma. Matrix extracts = PH46 and IS spiked into extract from blank plasma. Recovery = Mean extracted peak area/mean matrix extract peak area. n = replicates. n/a = not applicable.

### 3.4 Intra- and inter-assay accuracy and precision

The intra-batch accuracy and precision of the assay for PH46 in dog and rat plasma met the acceptance criteria for each of the three batches at each of the four concentrations assessed (Table 3). The inter-batch accuracy and precision also meet the acceptance criteria on each occasion.

**Table 3:** Intra- and inter-assay accuracy and precision for PH46 in dog and rat plasmas (results expressed as peak areas).

		10 ng/mL*			25 ng/mL*			300 ng/mL*			8000 ng/mL*		
	Occasion	1	2	3	1	2	3	1	2	3	1	2	3
PH46 in dog plasma	Replicate	n=6	n=6	n=6	n=6	n=6	n=6	n=6	n=6	n=6	n=6	n=6	n=6
	Intra-assay Mean	9.69	10.1	8.93	23.9	25.4	23.6	300	314	327	7830	8110	8260
	Intra-assay precision (%)	10.0	9.0	7.0	12.5	3.2	2.9	7.5	2.6	1.4	13.3	4.1	4.4
	Intra-assay accuracy (%)	96.9	101.0	89.3	95.6	101.6	94.4	100.0	104.7	109.0	97.9	101.4	103.3
	Inter-assay Mean	9.56			24.3			314			8070		
	Inter-assay precision (%)	9.7			7.8			5.6			8.1		
	Inter-assay accuracy (%)	95.6			97.2			104.7			100.9		
PH46 in rat plasma	Replicate	n=6	n=6	n=6	n=6	n=6	n=6	n=6	n=6	n=6	n=6	n=6	n=6
	Intra-assay Mean	8.60	9.18	10.1	26.1	25.5	25.9	311	332	319	8070	9130	9020
	Intra-assay precision (%)	12.6	10.4	6.2	5.6	3.8	2.2	0.9	5.5	1.6	3.3	5.5	8.2
	Intra-assay accuracy (%)	86.0	91.8	101.0	104.4	102.0	103.6	103.7	110.7	106.3	100.9	114.1	112.8
	Inter-assay Mean	9.28			25.8			321			8740		
	Inter-assay precision (%)	11.3			4.0			4.5			8.0		
	Inter-assay accuracy (%)	92.8			103.2			107.0			109.3		

* Nominal concentration of PH46.

### 3.5 Assay carry-over and matrix dilution

No significant carry-over was observed in the matrix blank and solvent samples injected after a ULOQ sample for PH46 or IS in both dog and rat plasma. The accuracy and precision for samples prepared at 80000 ng/mL and diluted at 10- and 100-fold met the acceptance criteria for dog and rat plasma (Table 4). These results showed that 10- and 100-fold dilutions of samples in control dog or rat plasma had no effect on the accuracy and precision of the method.

**Table 4:** Effects of dilution of PH46 with dog and rat plasmas (results expressed as ng/mL).

Dilution of PH46 with dog plasma			Dilution of PH46 with rat plasma	
Dilution factor	10	100	10	100
Replicate	n=6	n=6	n=6	n=6
Mean	68200	73500	74600	73700
Precision (%)	13.2	2.8	3.8	7.7
Accuracy (%)	85.3	91.9	93.3	92.1

### 3.6 Stability experiments

Data from all the stability test for both dog and rat plasma are shown in Table 5. All results met the acceptance criteria and PH46A and IS were stable in dog and rat plasma under current stability test conditions. It was concluded that PH46 and IS are stable in dog and rat plasma extracts under the following conditions: at least three freeze/thaw cycles at −20 °C, at least 24 h at ambient temperature, at least 31 days and 30 days, respectively at −20 °C, and 143.5 h and 243.5 h respectively at the auto-sampler temperature of at 4 °C before being re-injected. The mean re-injection stabilities of PH46 in extracts of dog and rat plasma stored at 4 °C for 131.5 h and 129.5 h respectively, also met the acceptance criteria.

**Table 5:** Stability tests of PH46 in dog & rat plasma (results expressed as ng/mL).

	PH46 concentration in dog plasma				PH46 concentration in rat plasma			
	25*		8000*		25*		8000*	
**a.**	Baseline 3 Cycles		Baseline 3 Cycles		Baseline 3 Cycles		Baseline 3 Cycles	
Replicate (n=6)								
Mean	23.6	25.5	8260	8000	25.5	26.4	9130	8650
Precision (%)	2.9	3.6	4.4	4.0	3.8	3.1	5.5	9.1
Accuracy (%)	94.4	102.0	103.3	100.0	102.0	105.6	114.1	108.1

**b.**	Baseline	24 h	Baseline	24 h	Baseline	24 h	Baseline	24 h
Replicate (n=6)								
Mean	23.6	25.1	8260	7670	25.5	27.7	9130	91205.
Precision (%)	2.9	4.3	4.4	5.2	3.8	6.4	5.5	5.1
Accuracy (%)	94.4	100.4	103.3	95.9	102.0	110.8	114.1	114.0

**c.**	Baseline	31 days	Baseline	31 days	Baseline	30 days	Baseline	30 days
Replicate (n=6)								
Mean	23.6	25.1	8260	7900	25.5	25.4	9130	8420
Precision (%)	2.9	10.6	4.4	2.3	3.8	4.4	5.5	6.6
Accuracy (%)	94.4	100.4	103.3	98.8	102.0	101.6	114.1	105.3

**d.**	Baseline	143.5 h	Baseline	143.5 h	Baseline	243.5 h	Baseline	243.5 h
Replicate (n=6)								
Mean	25.4	25.1	8110	7630	26.1	26.1	8070	7410
Precision (%)	3.2	10.6	4.1	6.4	5.6	5.9	3.3	4.0
Accuracy (%)	101.6	100.4	101.4	95.4	104.4	104.4	100.9	92.6

**e.**	10*	25*	300*	8000*	10*	25*	300*	8000*
Replicate (n=6)								
Mean	9.75	25.2	296	7500	8.71	25.4	318	8310
Precision (%)	11.6	11.9	7.2	13.7	11.0	7.9	1.8	5.3
Accuracy (%)	97.5	100.8	98.7	93.8	87.1	101.6	106	103.9

*Nominal concentration of PH46.

a. Freeze/thaw stability test; b. Ambient temperature stability test; c. Short term frozen matrix storage stability test; d. Autosampler stability (plasmas extract stored at 4 °C); e. Reinjection stability.

### 3.7 Pharmacokinetic study

No adverse reactions to treatment were observed when dogs 001 M and 002 M were dosed orally, while animal 003 M produced some frothy vomit at about 0.5 h post-dose and further yellow liquid vomit at 1.5 h post-dose. The animals were fed *ca* 2 h post-dose and after this period no further vomiting was observed. Except for these incidences of emesis, there were no other reactions to treatment.

**Fig. 5:**
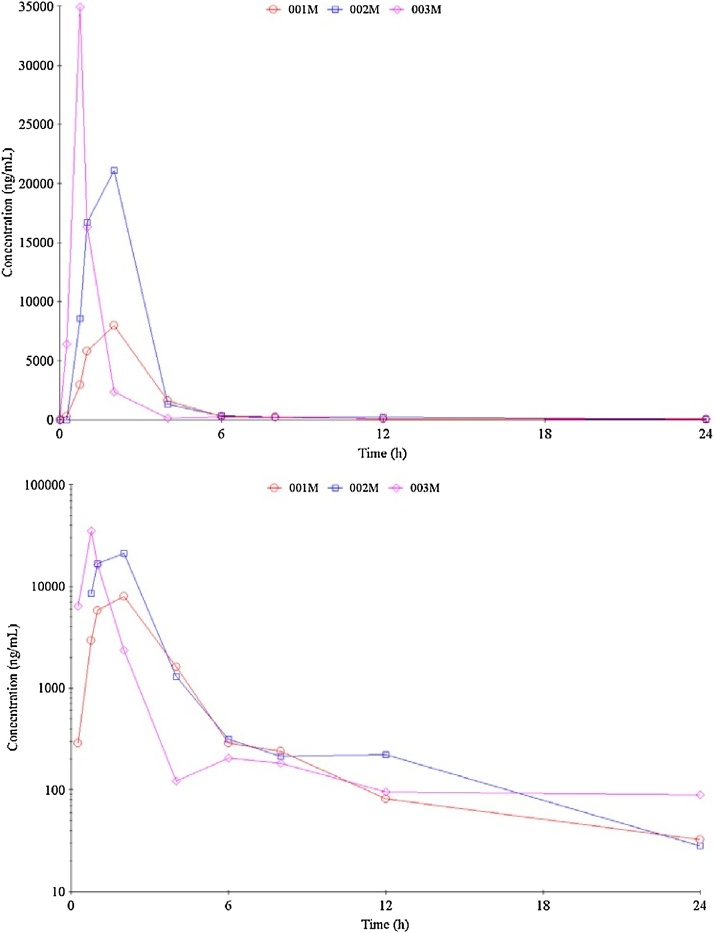
The plasma concentration profiles (upper: liner plot & lower: semi-log plot) of PH46 in male beagle dog following oral capsule administration of 100 mg/kg PH46A.

The pharmacokinetic parameters are presented in Table 6 and the pharmacokinetic profiles are represented graphically in Fig. 5. Maximal concentrations observed were variable within the 3 animals, with the highest concentration (C_max_) observed in animal 003 M (34900 ng/mL). T_max_ was 2 h for animals 001 M and 002 M, however, T_max_ for 003 M was 0.75 h. The half-life of elimination (T_1/2_) was similar in animals 001 M and 002 M (003 M not measurable) with a mean of 5.4 h. The mean AUC_(0-t)_ was 35000 ± 14700 ng.h/mL and mean AUC _(0-inf)_ was similar at 36900 ng.h/mL (n = 2).

**Table 6:** Pharmacokinetic parameters of PH46 in male beagle dog plasma following PH46A oral capsule administration of 100 mg/kg.

Dog	C_max_ (ng/mL)	C_max_/D (ng/mL)/ (mg/kg)	T_max_ (h)	AUC_(0-t)_ (ng.h/mL)	AUC_(0-t)_ /D (ng.h/mL)/ (mg/kg)	AUC_(0-inf)_ (ng.h/mL)	AUC_(0-inf)_ /D (ng.h/mL)/ (mg/kg)	T_1/2_ (h)
001M	7960	79.6	2.00	22200	222	22000	224	5.72
002M	21100	211	2.00	51100	511	51000	513	5.03
003M	34900	349	0.750	31700	317	n/r	n/r	n/r
n	3	3	3	3	3	2	2	2
Mean*	21300	213	2.00	35000	350	36900	369	5.38
SD	13500	135	-	14700	147	-	-	-

*Median for T_max_. n/r = Not reported, the coefficient of determination < 0.800.

## 4 Conclusion

A sensitive and specific analytical method was developed and validated for the determination of PH46 in dog and rat plasma. A range of testing, including linearity, accuracy, precision, specificity, matrix effect, carry-over, dilution effect and stabilities, were established for both matrices. The method meets EMA validation criteria. This method was successfully applied to a pre-clinical pharmacokinetic study in dog.

## Funding & Acknowledgement

This work was supported by The Wellcome Trust (grant reference no. 067033/Z/02/A), Enterprise Ireland and Charles River Laboratories (UK).

## Declaration of Competing Interest

All authors declare no conflicts of interest.
